# Interface Chemistry of Graphene/Cu Grafted By 3,4,5-Tri-Methoxyphenyl

**DOI:** 10.1038/s41598-020-60831-8

**Published:** 2020-03-05

**Authors:** Gina Ambrosio, Giovanni Drera, Giovanni Di Santo, Luca Petaccia, Lakshya Daukiya, Anton Brown, Brandon Hirsch, Steven De Feyter, Luigi Sangaletti, Stefania Pagliara

**Affiliations:** 1 I-LAMP and Dipartimento di Matematica e Fisica, Università Cattolica del Sacro Cuore, Via dei Musei 41, 25121 Brescia, Italy; 2 Division of Molecular Imaging and Photonics, Department of Chemistry, KU Leuven, Celestijnenlaan 200F, 3001 Leuven, Belgium; 3 Elettra Sincrotrone Trieste, Strada Statale 14 km 163.5, 34149 Trieste, Italy

**Keywords:** Chemistry, Condensed-matter physics

## Abstract

Chemical reaction with diazonium molecules has revealed to be a powerful method for the surface chemical modification of graphite, carbon nanotubes and recently also of graphene. Graphene electronic structure modification using diazonium molecules is strongly influenced by graphene growth and by the supporting materials. Here, carrying on a detailed study of core levels and valence band photoemission measurements, we are able to reconstruct the interface chemistry of trimethoxybenzenediazonium-based molecules electrochemically grafted on graphene on copper. The band energy alignment at the molecule-graphene interface has been traced revealing the energy position of the HOMO band with respect to the Fermi level.

## Introduction

The possibility to steer the band structure of graphene without a strong degradation of the ultrahigh mobility of the charge carriers and the destruction of its basic electronic properties is a main challenge of the current research on graphene. In pristine graphene, in fact, the Fermi energy separates the occupied and empty states at the Dirac point thus making it a gapless semimetal with a low density of states at the Fermi level. Due to the small conductivity of graphene in this condition, various approaches are being explored to change the charge carrier concentration such as the direct doping of graphene through the chemical modification of graphene itself, either by the introduction of defects or by elemental substitution of the carbon or through molecular adsorption. At the same time, an electronic bandgap for graphene, mandatory to develop graphene-based electronic devices, can also emerge as a result of the interaction with the substrate or the introduction of atomic doping, as well as of the presence of organic molecules on the graphene layer^1–3^.

Being simple and scalable, chemical modification is becoming a promising approach to modify the graphene electronic structure^4–7^. Nowadays, methods of graphene chemical modification includes the covalent attachment (or grafting) of aryl groups onto the graphene surface, which transforms the sp^2^ carbon atoms into the sp^3^ hybridization state^8,9^. With respect to the adsorption (non-covalent bonding) of the organic molecules on the graphene layer, the functionalization by covalent bonding is efficient in the graphene electronic structure modification^1^.

Based on the previous experimental and theoretical experiences with fullerene and carbon nanotubes, (electrochemical) reduction of diazonium ions shows to produce highly reactive free radicals which attack the sp^2^ carbon atoms of graphene forming a covalent bond. The reduction reaction consists of an electron transfer to the aryl diazonium ions, releasing molecular nitrogen and creating a reactive intermediate aryl radical. This radical usually reacts directly with the carbon surface or can undergo polymerization with other aryl radicals. Electron transfer to diazonium ions can be spontaneous or can be controlled electrochemically (electrons are transferred to aryldiazonium ions from the carbon network by an external electrical field). To date, a lot of different diazonium salts have been used for graphene modification^4,6,9^.

The Gr/Cu (graphene/Cu) interface is extensively studied and is among the candidates for scaling up the production of graphene. In this context the evaluation of the energy level alignment between the organic layer and graphene is of paramount importance to develop hybrid junctions. This feature makes the overall system interesting to develop devices based on a metal/Gr/organic layer interface where the covalently bonded diazonium-derived molecules can interact with the environment and transfer charges to the graphene layer beneath.

It is important to note that the covalent attachment of aryl groups onto the graphene surface requires the use of an electrolyte solution, containing acids and salts, that makes this technique potentially exposed to contaminations that could in principle affect the properties of graphene. In order to assess the consequences of electrochemically assisted grafting on the graphene electronic structure, a systematic study of the electronic properties across the different stages of sample preparation and grafting is mandatory.

**Figure 1:**
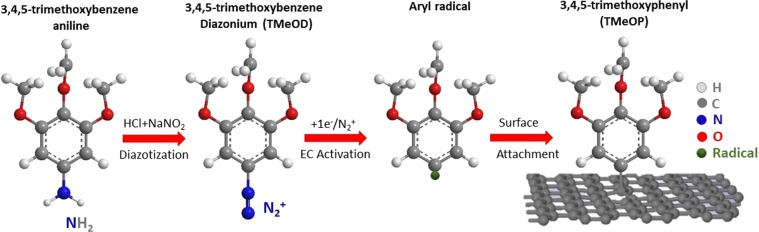
Scheme showing *in situ* formation and grafting of aryl radical. The aniline precursor is converted into a diazonium ion which is then electrochemically activated (EC) to form the aryl radical.

In the present study, graphene has been grafted by *in situ* generated 3,4,5-trimethoxybenzenediazonium (TMeOD) cations. This compound is expected to form aryl radicals via electrochemical reduction and to graft the graphene as 3,4,5-trimethoxyphenyl (TMeOP) units^10^ (Fig. 1). For simplicity, we refer to it as TMeOP-grafted graphene. The grafting of TMeOP has been carried out on graphene grown on copper foil, as well as on graphene grown on Cu (111) (see Methods and Supporting Information for details).

The nature of the chemical modification of graphene, the relation between molecular structure and film morphology have been deeply investigated in literature^8,11,12^, however a systematic study of the electronic properties to understand the chemical environment is still missing. This study is a standard procedure for functionalized graphene in UHV condition as usually happened for a non-covalent approach^13^. To unambiguously reach this result, we have also prepared control samples where the Gr/Cu interface was treated in the electrochemical cell by cycling the potential in a solution of HCl and NaNO_2_ without aniline precursors. These samples are hereafter denoted as treated samples.

The chemically modified graphene has been characterized through Raman spectroscopy. Then a detailed study of both core levels and valence band has been carried out by comparing the photoemission spectra collected from the pristine, from the treated and from TMeOP-grafted graphene. In order to single out the contribution of the grafted molecular units to the core-level photoemission spectra, the data of the C 1 s, O 1 s, and N 1 s core levels have been contrasted and compared to those collected from graphene exposed to *in situ* generated *3,5*-bis-*tert*-butylbenzenediazonium (TBD) cation, leading to the grafting of *3,5*-bis-*tert*-butylphenyl (TBP) units, as well as from grafted Highly Oriented Pyrolytic Graphite (HOPG). TBD has been chosen as it does not contain oxygen, allowing for an unambiguous discussion of the oxygen contribution to the measured spectra. Indeed, oxygen may have a three-fold origin, i.e. from the growth solution (water and NO_2_), from unreacted TMeOP, but also from the Gr/Cu interface. Finally, the band structure at the Γ and K points of the Brillouin zone has been experimentally probed by angle-resolved photoemission spectroscopy (ARPES) with synchrotron radiation. Supported by density functional calculations on 3,4,5-trimethoxybenzene, as well as on TMeOP-grafted on free-standing graphene the analysis of the density of states in the valence band region has also allowed us to identify the HOMO band of TMeOD and set its energy with respect to the Fermi level, thus obtaining a scheme for the band alignment at the Gr/TMeOP hybrid interface.

## Results and Discussion

**Figure 2:**
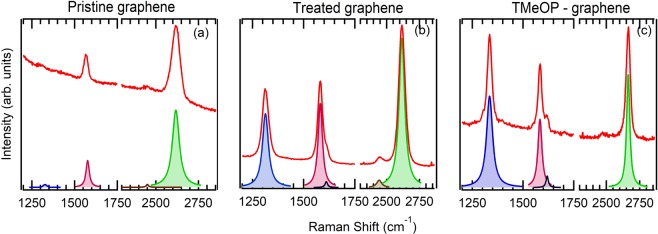
Raman of (**a**) pristine graphene on copper foil, (**b**) treated graphene on copper foil, (**c**) 5 mM of TMeOP-grafted graphene on copper foil. “Treated” refers to graphene exposed to grafting conditions, however in absence of TMeOD.

Raman spectroscopy was carried out to investigate the influence of chemical modification on the graphene structural properties (Fig. 2). The Raman spectrum of pristine graphene mainly displays two main peaks denoted as G and 2D. The G band, located at 1580 cm^−1^, is associated with in plane C-C stretching of sp^2^ hybridized carbon atoms in the planar graphene skeleton^7,14,15^. The 2D peak is associated with in plane breathing mode of carbon atoms and is a second order Raman scattering process (Fig. 2a). The D band located at 1320 cm^−1^ is associated with defects in the regular network of sp^2^ hybridized carbon atoms and the presence of sp^3^ hybridized carbon atoms. The D/G band intensity ratio can be used to compare and qualitatively estimate the defects and the degree of disorder in the pristine and grafted graphene. For pristine graphene on Cu, the I_D_/I_G_ ratio is 0.105 (Fig. 2a); after the treatment (in absence of TMeOD), the D band intensity increases and the ratio I_D_/I_G_ becomes 0.92, suggesting that some defects in the regular network of sp^2^ hybridized carbon atoms are introduced by this procedure (Fig. 2b). The I_D_/I_G_ ratio further increases when molecules are grafted, up to a value of 1.40 for TMeOP-grafted graphene. At the same time, the shoulder D’-band appears, confirming that the defects related to sp^3^ hybridization due to the grafting procedure (Fig. 2c) significantly increases.

After the grafting procedure, core level photoemission spectroscopy measurements were carried out using X-ray photoelectron spectroscopy (XPS) (a) on the pristine graphene on Cu foil (Gr/Cu), (b) on the treated Gr/Cu foil, and (c) on Gr/Cu foil grafted with TMeOP units. XPS spectra have been collected also on (d) 5 mM of TBP-grafted graphene on Cu foil for comparison. The TBD units has been chosen because, as shown in Supplementary Information, the methoxy (O-CH_3_) groups of the TMeOP units are substituted by C_4_H_9_ groups where the oxygen is missing, allowing to better single out the different contributions in the carbon and oxygen core levels.

The wide scan photoemission spectra (See Supplementary Information, Fig. S3) collected on Gr/Cu foil, treated Gr/Cu foil and on the two grafted Gr/Cu foil, i.e. grafted with TMeOP and TBP, show all the features ascribed to carbon, copper and oxygen atom.

**Figure 3:**
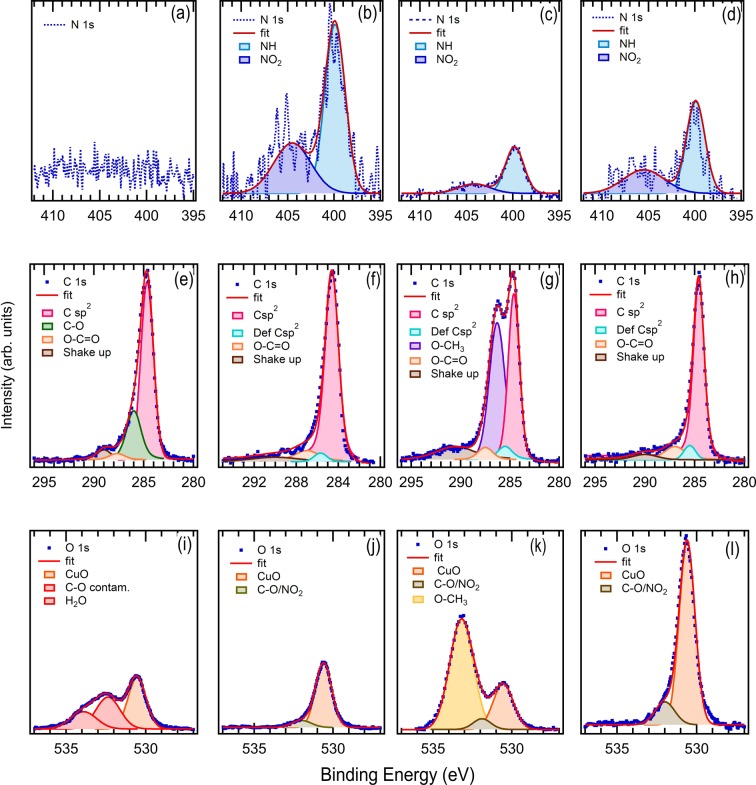
N 1 s core level XPS spectra of (**a**) pristine Gr/Cu, (**b**) treated Gr/Cu, (**c**) TMeOP-grafted on Gr/Cu foil and (**d**) TBP-grafted on Gr/Cu foil. C 1 s core level XPS spectra of **(e**) pristine Gr/Cu foil, (**f**) treated Gr/Cu foil, (**g**) TMeOP-grafted on Gr/Cu foil and (**h**) TBP-grafted on Gr/Cu foil. O 1 s core level XPS spectra of (**i**) pristine Gr/Cu foil, (j) treated Gr/Cu foil, (**k**) TMeOP-grafted on Gr/Cu foil and (**l**) TBP-grafted on Gr/Cu foil. The intensity of the N1s and O1s core levels is scaled consistently with the quantification reported in Table 1.

In Fig. 3, the C 1 s and O 1 s XPS peaks are shown, along with the N 1 s core level. Performing several scans around a binding energy BE = 400 eV^16^, in fact, a very small feature, ascribed to N 1 s, appears in the XPS spectra of the treated Gr/Cu foil, the TBP-grafted, and TMeOP-grafted samples. As N_2_ is released by the electrochemically induced formation of the radical species^17^, none of the two molecules is expected to contain nitrogen atoms after the reduction procedure.

To clarify the origin of N 1 s feature, we have performed the quantification of the atomic elements, summarized in Table 1. A low, but detectable, amount of N with respect to the dominant C signal is found in the treated (N/C=0.04) and in the grafted Gr/Cu foil (N/C=0.01) samples. The presence of N 1 s in the treated sample suggests that the origin of the N 1 s peak could be attributed to NaNO_2_ employed for the grafting process. The N 1 s core level spectra are shown in Fig. 3a–d, where the treated Gr/Cu foil sample is compared with TMeOP and TBP-grafted Gr/Cu foil; we note, in fact, that no feature ascribed to N 1 s appears in the pristine Gr/Cu foil sample.

**Table 1:** Quantification of elements (N, O, C) and N/C and O/C ratios obtained from the analysis of the XPS wide range spectra of pristine Gr/Cu foil, Treated Gr/Cu foil, TMeOP and TBP-grafted molecules.

Sample	N 1 s (%)	O 1 s (%)	C 1 s (%)	N/C	O/C
Pristine Gr/Cu	–	23.8	76.2	–	0.31
Treated Gr/Cu	3.3	12.5	84.2	0.04	0.15
TMeOP-grafted	0.7	21.8	77.5	0.01	0.28
TBP-grafted	1.2	34.3	64.5	0.02	0.53

The spectrum of the N 1 s core level can be interpolated by two Lorentzian peaks ascribed to the amine nitrogen group (N-H) at 399.5 eV^18^, and to nitrogen of nitro group present in the form of N-O_2_ bonds with an energy maximum at 405 eV^19,20^. The N 1s lineshape is preserved in TBP and TMeOP-grafted samples. The possibility of a N 1s signal arising from unreacted diazonium salt can be ruled out as such signal would produce two peaks at 403.8 and 405.1 eV, which are not observed^21,22^. Therefore, we can assert that the observed N-groups are released from the growth solution (aqueous solution of NaNO_2_ and H_2_O) and they can be ultimately found anchored to the low sp^2^ defects of graphene lattice created by the electrochemical process or they can intercalate between graphene and copper.

The C 1 s spectra are shown in Fig. 3e–h. The main signal of C 1 s for the pristine Gr/Cu foil, treated Gr/Cu foil and TBP-grafted Gr/Cu foil is dominated by the carbon at 284.6 eV due to graphene sp^2^ bonding^23,24^.

In addition, other structures appear at larger BE for the treated sample. Two features can be ascribed to the graphene π−π* satellite and to the oxygen contamination (O-C=O contribution^4,9^).

For the TMeOP-grafted Gr/Cu foil, the presence of the methoxy contribution (O-CH_3_ feature) in the C 1 s confirms the success of the EC grafting procedure and suggests that the graphene coverage by TMeOP molecules is very high. We estimate, as shown in the supplementary, a coverage of about 1 molecule per 3 graphene unit cells. This result is in agreement with the increase of the D peak in the Raman spectrum if we consider the high density defect regime^25^ ascribed to the grafted molecules. In this approximation, we obtained, in fact, L_D_=1.07 nm which corresponds to a molecular density of 1 molecule per 4.5 ± 0.5 graphene unit cells in agreement with the coverage estimated by XPS measurements.

Consistently, a further structure, at a binding energy of 285.4 eV, due to C sp^3^ hybridization has been added in the C1s core level of the grafted samples. For the TBD-grafted, the area of the sp^3^ contribution in the C 1s core level (Fig. 3h) has been considered the same of the TMeOP-grafted (Fig. 3g) because the concentration of molecules in the growth solution was for both sample 5 mM.

In the treated graphene, without grafted molecules, we can assume that the defect density, ascribed to carbon atoms out of strictly sp^2^ configuration, is low in agreement with STM measurements (See Supplementary Information for details, Fig. S4). The greatest quantity of nitrogen in this sample with respect to the grafted samples could be due to the capability of these defects to partially accommodate N-groups or with the intercalation of nitrogen between graphene and copper.

The analysis of oxygen is more complicated (Fig. 3i–l). The effect of atmospheric adsorbates on graphene was found to be relevant, as it can improve or degrade the carrier mobility depending on the nature of the impurities^26^. In Table 1, it is possible to observe for example how the O 1 s amount significantly changes with the sample type. The O/C ratio, last column of Table 1, is smaller in TMeOP-grafted rather than in TBP-grafted, even if the O quantity is expected to be larger in TMeOP-grafted molecules due to the O-CH_3_ groups. This discrepancy will be clarified by discussing the origin of each peak contributing to the O 1 s spectral weight. In the pristine Gr/Cu, three structures dominate the O 1 s core level, the contributions at higher binding energy (about 532.4 and 533.9 eV) are related to the graphene surface contamination due to the air exposure and it is generally ascribed to C-O and H_2_O groups. The electrochemical process seems to mainly eliminate these oxygen contamination components. This result is in agreement with the literature^27^, where the electrochemical process is usually adopted to reduce the graphene oxide. Traces of this contamination persist in the treated and grafted samples as proved by the presence of the O-C=O feature.

The contribution, at lower binding energy (at about 530.5 eV) in all the samples can be ascribed to the oxygen trapped between the graphene and the copper substrate^7,21,28–30^.

To confirm this interpretation, we have verified that when the XPS measurement is more surface sensitive, by changing the analyzer take-off angle from the normal emission (90° with respect to the sample surface, more bulk sensitive) to the take-off angle of 30° (more surface sensitive), the contribution of the oxygen contamination doubles (See Supplementary Information, Fig. S6).

With respect to the treated Gr/Cu sample, in the TMeOP-grafted sample a new feature at about 533 eV^31^ is detectable, which is therefore ascribed to the methoxy (O-CH_3_) groups. As shown in the supplementary information (Fig. S5), this feature increases with the molecule concentration in the solution and with the feature ascribed to the O-CH_3_ group in the C 1 s and O 1 s XPS spectra.

To further discuss the origin of the contribution at 530.5 eV to the O 1 s XPS spectrum and to verify whether it comes from the CuO_x_ layer at the interface between graphene and copper foil, we have collected the O 1 s core levels also on TMeOP-grafted on HOPG (See Supplementary Information, Fig. S7). Indeed, this peak is missing in the grafted HOPG as the O 1 s XPS spectrum shows only the contributions from oxygen contamination or from the O-CH_3_ group at higher BE.

The presence of a CuO_x_ layer between Gr and Cu may have an impact on the extent of the Gr-Cu coupling. As it is reported in literature^27^, when graphene is grown on copper, a charge transfer from Cu surface to graphene takes place resulting in n-type doping of graphene and shifting of Dirac point 0.38 eV below the Fermi level. On the contrary, when graphene grows directly on copper oxide, it becomes electronically decoupled from the substrate and shows properties comparable to the freestanding graphene with the Dirac point located at the Fermi level^32^.

**Figure 4:**
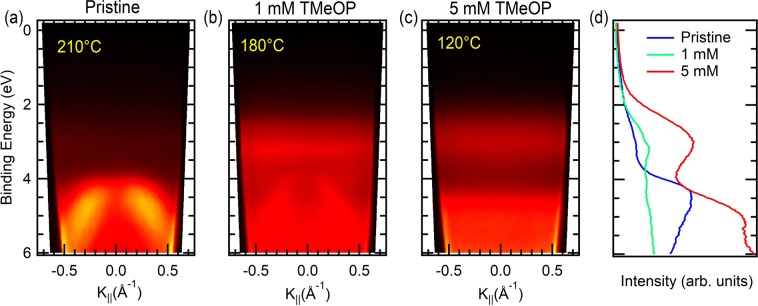
ARPES measurements in p polarization around the Γ point of the first Brillouin zone on (**a**) pristine Gr/Cu foil, (**b**) on 1 mM TMeOP-grafted on Gr/Cu(111), (**c**) on 5 mM TMeOP-grafted on Gr/Cu foil, and (**d**) corresponding energy distribution curves (EDCs) at k_||_ = 0 Å^−1^. The annealing temperature carried out before the ARPES acquisitions is also reported.

In this scenario, we can assume that, in our case, TMeOP units are grafted on a graphene layer whose properties are intermediate between freestanding graphene and doped graphene, as expected for the weakly interacting graphene on copper. This is also confirmed by ARPES measurements collected around the Γ point of the Brillouin Zone (BZ) for pristine Gr/Cu and TMeOP-grafted on Gr/Cu at different molecular concentrations (Fig. 4). In the former, the d band of Cu appears between 2 and 4 eV^32^, while the σ band of graphene dominates the spectra at BE= 4 eV (Fig. 4a). When graphene is grafted by TMeOP units a new, dispersion-less, structure appears at BE=3.05 eV below the Fermi level which can be ascribed to the TMeOP HOMO state (Fig. 4b–d). The lack of dispersion indicates that the electrons photoemitted from TMeOP-grafted originate from localized molecular-like electronic states, rather than from k-dispersing electronic bands. At the K point of the BZ, the Dirac point of our pristine Gr/Cu appears downshifted less than 100 meV with respect to the Fermi level after an annealing temperature of 200 °C–350 °C (See Supplementary Information, Fig. S8). It is known that graphene grown on metallic copper is n-type doped with the Dirac point shifted by about 380 meV below the Fermi level^32^, while on oxidized Cu the graphene is decoupled from its metallic substrate due to the oxide layer and therefore it is undoped with the Dirac point at the Fermi level. In our case, the Dirac point shifted about 100 meV below the Fermi level in the pristine sample as reported by A. J. Marsden *et al*.^33^, confirms the presence of a partially oxidized copper layer at the interface able to partially decouple graphene from the metallic substrate^34^.

The ARPES spectra on the 5 mM TMeOP-grafted Gr/Cu foil (Fig. 4c) at the Γ point is completely dominated at low binding energy by the HOMO at about 3 eV and HOMO-1 bands at 4.5–5.0 eV of TMeOP film. The absence of features ascribed to the graphene σ band (partially visible on the 1 mM TMeOP- Gr/Cu(111), Fig. 4b) at the Γ point and to the Dirac cone at the K point (see Supplementary Information Fig. S8) confirms the high quantity of grafted molecules that completely covers the graphene surface. Referring to Fig. 4d we can conclude that the HOMO of the TMeOP/Gr/Cu lays about 1 eV above the σ bands of pristine graphene.

**Figure 5:**
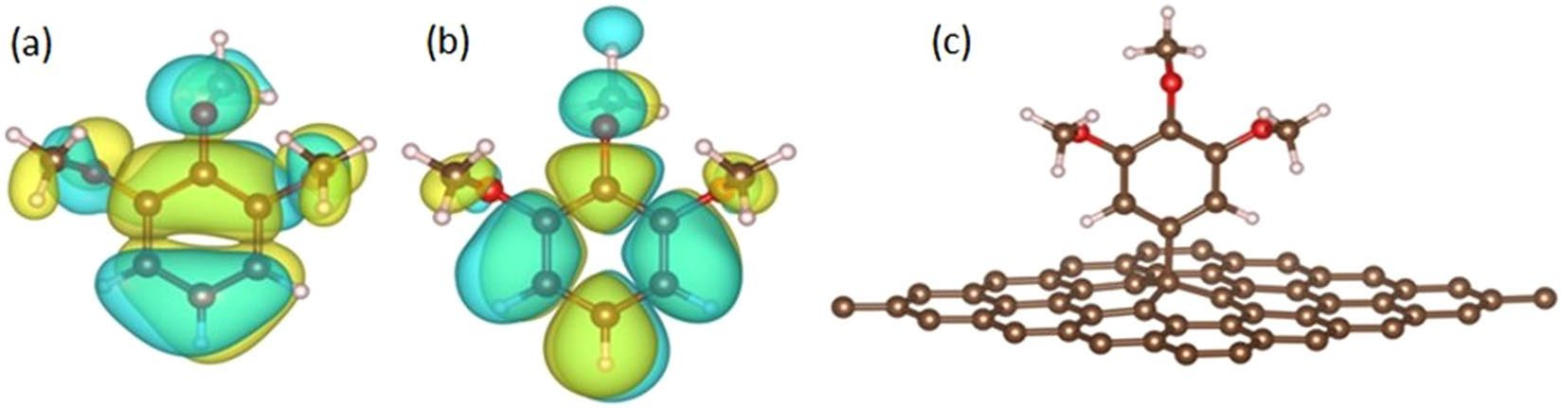
Schematic atomic structure for DFT calculations: (**a**) calculated real part of HOMO and (**b**) LUMO of 3,4,5-trimethoxybenzene; (**c**) relaxed unit cell for the TMeOP grafted graphene. Brown circles are for carbon atoms, red for oxygen and white balls for hydrogen.

In order to understand the effects of TMeOP grafting on graphene, we carried out a set of several ab-initio simulations. At first, we considered the isolated 3,4,5-trimethoxybenzene (TMeOB), as shown in Fig. 5a,b. The calculated HOMO-LUMO gap is 4.70 eV; as compared to the HOMO, the molecule LUMO is strongly depleted on oxygen atoms, which tend to acquire electrons from the neighbouring C and H atoms.

Then, free-standing graphene and TMeOP-grafted graphene, have been considered (Fig. 5c). In these calculations, the molecule is bond over a 5 × 5 graphene supercell, consisting of 50 carbon atoms; the TMeOP density is thus 0.76 molecule/nm^2^.

**Figure 6:**
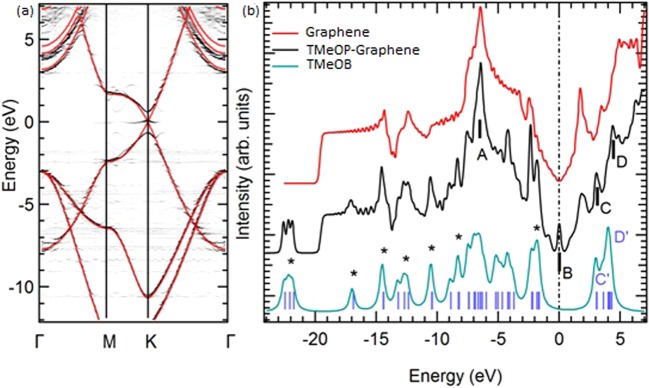
(**a**) Calculated band structure for graphene (red) and TMeOP-grafted graphene (grey, folded on the graphene unit cell); (**b**) calculated DOS spectra for graphene, isolated 3,4,5-trimethoxybenzene (TMeOB) and grafted graphene. TMeOP pseudo DOS has been obtained by convoluting the DFT eigenvalues (blue vertical lines) with gaussian peaks.

The grafting induces structural and electronic modifications. The graphene is strongly buckled with respect to the unrelaxed, free-standing, graphene plane the sp^3^ C atom and its three C neighbours are lifted of about 0.581 Å and 0.174 Å, respectively. Figure 6a displays the band structure of free-standing graphene (thick red lines) and of the TMeOP-graphene supercell (grey lines), while the corresponding calculated DOS are shown in Fig. 6b.

We note that the electronic structure calculations predict a weak peak (B) in the density of states (DOS) at the Fermi level. The origin of this in-gap state is mainly due to the breakdown of the lattice symmetry of graphene when grafted by TMeOP units and to the change in carbon hybridization from sp^2^ to sp^3^ at the grafting site. In fact, consistent with literature^35^, a change in carbon hybridization from sp^2^ to sp^3^ in the graphene layer introduces a flat band at the Fermi level, yielding a small peak in the DOS. This state is not present in ARPES measurements. We ascribe this discrepancy to disorder effects that are not accounted for in the supercell calculations. The DFT calculations are in fact carried out considering an ordered structure formed by a TMeOP units bound over a 5 × 5 graphene supercell, as shown in Fig. 5. In the measured sample, on the contrary, the molecules, with an estimated density of about 1 molecule per 3 graphene hexagons, are randomly arranged on the graphene layer without any detectable periodicity. This disorder may therefore quench the intensity of the in-gap state expected from the supercell calculations.

In spite of the electronic hybridization between the molecule and graphene, it is possible to easily pinpoint the TMeOB contribution in the integral density of states (DOS) spectra, shown in Fig. 6b. Several features, labelled with an asterisk, can be recognized in 3,4,5-trimethoxybenzene (blue in Fig. 6b) and TMeOP-graphene system (black) spectra. Accordingly, the resulting HOMO for the grafted molecule should be at BE = 1.8 eV, while the peak ascribed to the σ band is found at about 3 eV. In agreement with the experiments (Fig. 4d), the relative position of the HOMO with respect to the graphene σ band is about 1 eV. A difference exists between the calculated and the experimental absolute energy values of the HOMO and σ band, this can be ascribed to the approximation of the calculation. In the DFT calculation the σ band appears at 3 eV rather than at 4 eV already in the graphene, having considered, for simplicity, a free-standing graphene layer rather than graphene on Cu or better on CuO_x_/Cu. The other DOS feature ascribable to graphene, peak A in Fig. 6b, and the overall shape of the band dispersion (Fig. 6a) do not show any displacement with respect to the undoped case, apparently ruling out the presence of major energy shifts due to the grafting, again in agreement with our ARPES results on the pristine and 1 mM TMeOP-grafted graphene in Fig. 4a,b. An in-gap state contribution can be easily observed in the calculated DOS by the peak (labelled B) at the Fermi level which requires a more detailed ARPES investigation at the K point of the BZ. Finally, two additional DOS features in the empty states (peaks C and D) could be ascribed to TMeOP-grafting and should correspond to C’ and D’ peak of the isolated molecule; the interaction with graphene thus induces a small increase (about 0.4 eV) in the energy of TMeOB states close to the LUMO.

## Conclusions

The interface chemistry of graphene grown on copper and functionalized by covalent bonding with TMeOP units has been studied by core level and angle-resolved valence band photoemission. Two main effects can be ascribed to the electrochemical treatment, the application of the electric field creates a low density of defects in graphene that can accommodate N-groups, in addition the treatment eliminates the oxygen contamination on the top of the graphene layer.

Moreover, comparing the core level spectra collected on both TMeOP and TBP-grafted on graphene, the contribution of the methoxy groups has been singled out in C 1 s and O 1 s spectra. An extra oxygen contribution coming from a CuO_x_ layer between graphene and copper allows to regard graphene as almost decoupled from the metallic substrate and to measure the alignment between graphene σ and π bands and the HOMO of TMeOP, which is found to lay about 1 eV above the top of the σ bands, consistently with DFT calculations.

Therefore, despite the presence of the solution in the electrochemical cell, that makes this treatment a grafting procedure potentially exposed to contaminations from electrolytes, the results reported in this study show that the functionalization by diazonium chemistry does not affect significantly the chemical environment, and that the electronic properties at the interface are mainly determined by the covalently grafted molecules.

This finding demonstrates that high quality grafting can be achieved in a set-up with growth conditions less demanding with respect to UHV-related methods^36^, therefore disclosing the possibility to apply photoemission techniques to systems prepared on the basis of similar electrochemical methods^37^, and ultimately enabling a scalable pathway for the production of functionalized graphene layers.

## Methods

Electrochemical measurements were performed using an Autolab PGSTAT101 potentiostat (Metrohm Autolab BV, The Netherlands). The electrochemical modification procedure was carried out in a homemade single-compartment three–electrode cell with a working electrode area of 38.5 mm^2^, Pt wire counter and Ag/AgCl/3 M NaCl reference electrodes. 3,5-bis-tert-butylbenzenediazonium (TBD) chloride and 3,4,5-trimethoxybenzenediazonium (TMeOD) chloride are unstable and decompose rapidly: hence they were synthesized from the corresponding aniline precursor immediately prior to electrochemical reduction. This procedure involves 5 mL of a 1 mM (or 5 mM) 3,4,5-trimethoxyaniline (97%, Sigma-Aldrich) or 3,5-bis-tert-butylaniline (98%, Sigma-Aldrich) in 50 mM HCl (Sigma-Aldrich) aqueous solution which was mixed with 50 µL (or 250 µL) of aqueous NaNO_2_ (0.1 M) for activation of the diazotization reaction. Within 90 seconds, this mixture was gently shaken and pipetted into the EC cell. Cyclic voltammetry was used for the electrochemical activation. In the measurements the potential window was chosen from 0.3 V to −0.6 V, scan rate 0.1 V/s for 3 cycles. After modification, the TMeOP modified samples were rinsed with Milli-Q water (Milli-Q, Millipore, 18.2 MΩ cm, TOC <3 ppb) to remove any physisorbed material from the surface and dried in a stream of Argon. The STM measurements were acquired with a molecular imaging STM system operating in constant current mode. The tip was obtained by a Pt-Ir wire (80–20%, diameter 0.25 mm).

The substrates used are CVD graphene on Cu foil obtained from Graphenea, graphene on Cu(111) obtained from IMEC and HOPG (grade ZYB, Advanced Ceramics Inc., Cleveland, OH).

In order to avoid the detachment of the phenyl units, all the X-ray photoemission measurements have been collected after an annealing treatment in ultra-high vacuum conditions at temperatures lower than 393 K. Core level spectra have been collected with a properly calibrated^38^ VG-SCIENTA R3000 analyzer and the Al Kα line of a twin anode X-ray source, operating in ultra-high vacuum conditions (base pressure 2×10^−10^ torr).

Angle-resolved valence band spectra were carried out at the BaDElPh beamline of the Elettra synchrotron in Trieste (Italy) using a photon energy of 34 eV and an hemispherical electron analyzer SPECS Phoibos 150 with a 2D-CCD detector system^39^. The overall energy and angular resolution were set to 100 meV and 0.3°, respectively. All the ARPES maps were collected at room temperature (RT) after an annealing treatment. The annealing temperature (reported in the Figures), in this case, is the minimum value that allows to observe a clear ARPES map.

Raman measurements were performed with an OmegaScope 1000 (AIST-NT). Laser light (632.8 nm) from a He-Ne laser was focused onto the sample surface from the side (with an angle of 28° to sample surface) and top, for ‘grating’ and ‘normal’ measurements, respectively, through an objective (MITUTOYO, BD Plan Apo 100x, N.A. 0.7). Optical density at sample surface was about 500 kW/cm^2^. Raman scattering was collected with the same objective and directed to a Raman spectrograph (Horiba JY, iHR-320) equipped with a cooled-charge coupled device (CCD) camera operated at −100 °C (Andor Technology, DU920P-BRDD) through a pinhole, a dichroic mirror (Chroma Technology Corporation, Z633RDC) and long pass filter (Chroma Technology Corporation, HQ645LP). Accumulation time for each point in ‘grating’ measurement was 1 s. All of the measurements were carried out under ambient conditions and at room temperature.

The TMeOB, TMeOD-grafted graphene and pristine graphene electronic structures have been evaluated through ab-initio density functional theory calculations, in the framework of the GGA-PBE approximation^40^. Van der Waals corrections to the exchange-correlation potential have been neglected, due to the direct chemical bond induced by grafting. Calculations have been performed with the ABINIT package^41^ in the framework of Projector-Augmented wave atomic description. A large cut-off energy (700 eV) has been considered for the plane-wave basis definition. All atomic position have been relaxed up to a maximal interatomic force of 10^–5^ Ha/Bohr. For the graphene and the grafted graphene cells a 7 ×7 ×1 Monkhorts-Pack grid has been adopted, with an adequately large cell size on the out-of-plane direction (15 Å); for the isolated TMeOB, the molecule was placed in a wide (15 Å) cubic cell size, with a single k point. For each case, the convergence on the total energy was set to the 10^−9^ Ha level.

## Supplementary information

Supplementary information.
