# Supplementary material for: Interface Chemistry of Graphene/Cu Grafted By 3,4,5-Tri-Methoxyphenyl
Source: Sci Rep. 2020 Mar 5;10:4114. doi: 10.1038/s41598-020-60831-8 (PMC7058071; doi:10.1038/s41598-020-60831-8)
Supplement: Supplementary file 1 — Supplementary information. [file 41598_2020_60831_MOESM1_ESM.pdf]

Supplementary Information

INTERFACE CHEMISTRY OF GRAPHENE/Cu GRAFTED BY 3,4,5-TRI-METHOXYPHENYL

*Gina Ambrosio[1,2], Giovanni Drera[1], Giovanni Di Santo[3], Luca Petaccia[3], Lakshya Daukiya[2], Anton Brown[2], Brandon Hirsch[2], Steven De Feyter[2], Luigi Sangaletti[1], and Stefania Pagliara[1]**

[1] I-LAMP and Dipartimento di Matematica e Fisica, Università Cattolica del Sacro Cuore, Via dei Musei 41, 25121 Brescia, Italy

[2] Division of Molecular Imaging and Photonics, Department of Chemistry, KU Leuven, Celestijnenlaan 200F, 3001 Leuven, Belgium

[3] Elettra Sincrotrone Trieste, Strada Statale 14 km 163.5, 34149 Trieste, Italy

Figure S1 shows the structural formulas of the 3,4,5-trimethoxybenzenediazonium (TMeOD) and 3,5- bis-tert-butylbenzenediazonium (TBD) employed for the grafting procedure.

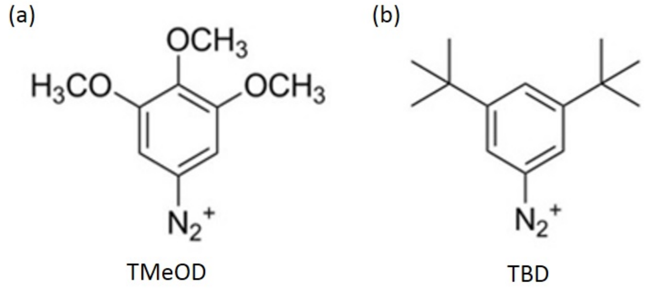

Figure S1: *Scheme of the molecules: (a) 3,4,5-trimethoxybenzenediazonium (TMeOD) and (b) 3,5-bis-tert-butylbenzenediazonium (TBD).*

Electrochemical surface modification of graphene was carried out during cyclic voltammetry. During the reduction process the diazonium molecules release the N_2 and they graft the graphene surface as 3,4,5-trimethoxyphenyl (TMeOP) and *3*,5-bis-*tert*-butylphenyl (TBP) units. Figure S2 shows cyclic voltammogram of treated graphene on copper, of 5 mM and 1 mM TMeOP-grafted units, and of 5 mM TBP-grafted units.

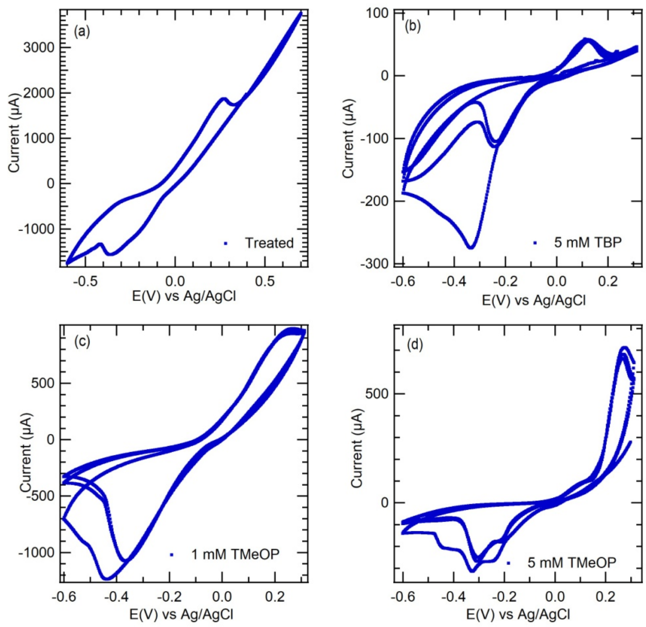

Figure S2: *Cyclic voltammetry of (a) treated Gr/Cu foil, (b) 5 mM TBP-grafted on Gr/Cu foil (c) 1 mM TMeOP-grafted on Gr/Cu(111) and (d) 5 mM TMeOP-grafted on Gr/Cu foil.*

In Figure S3, the wide core level photoemission spectra collected on Gr/Cu foil, treated Gr/Cu foil and on the two Gr/Cu substrates grafted with TMeOP-grafted on Gr/Cu foil and Gr/Cu(111) and TBP-grafted on Gr/Cu foil are shown. Three peaks ascribed to photoemission from Cu 3d, Cu 3p and Cu 3s levels of the copper substrate are detectable, along with the C 1s and O 1s core levels. The binding

energies (BE) of Cu 3s, Cu 3p and Cu 3d lines are 122.6 eV, 75.1 eV and 3.1 eV, respectively. For C 1s BE= 284.6 eV while for O 1s BE= 530.6 eV.

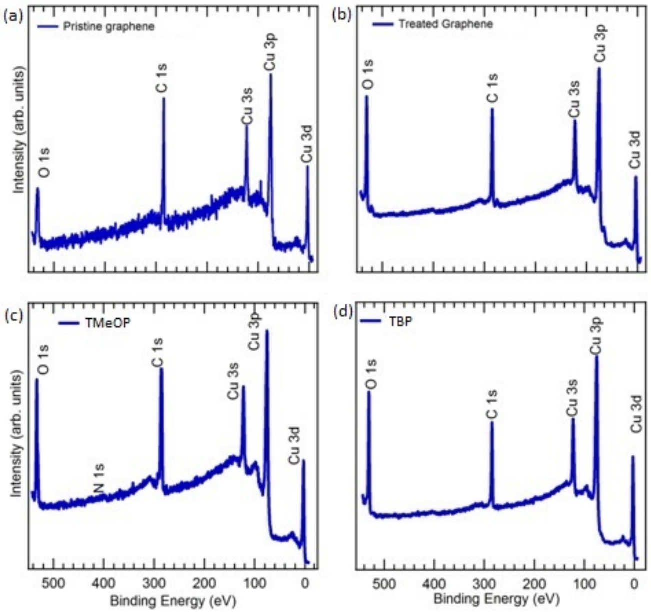

Figure S3: *Wide XPS spectra of (a) pristine Gr/Cu foil, (b) treated Gr/Cu foil, (c) 5mM TMeOP-, and (d) 5mM TBP-grafted Gr/Cu foil.*

From the analysis of the C 1s core level (Figure 3g of the manuscript) of TMeOP-grafted graphene, we have estimated the molecular coverage as density of the units per graphene unit cell, as reported in the following.

To evaluate the contribution of graphene (G), as first we estimate the contribution of the grafted molecule Q_{mol} from the integrated intensity of the methoxy group (O-CH_3) contribution divided by six, being six the number of C atoms related to the oxygen in in the molecule (i.e. 3 C atoms for the O-CH_3 group and 3 C in the ring). Q_{mol} then corresponds to the Q_{Csp^3} weight. Therefore, we have subtracted from the C sp^2 signal (Q_{Csp^2}) the sp^2 contribution coming from the benzene ring of the molecules. For the TMeOP units, this contribution is obtained multiplying by 3 the signal of Q_{mol}, as 3 out of 6 C atoms in the ring are sp^2 bonded.

$$G = Q_{Csp^2} - 3 \cdot Q_{mol}.$$

Then, to estimate the contribution of the number of graphene unit cells, we have summed the value of G and Q_{Csp^3} in order to obtain the contribution of all C atoms in the graphene layer. Then, as the number of C atoms in the unit cell of graphene is 2, the number of graphene cells (N_{cell}) is:

$$N_{cells} = (G + Q_{Csp^3})/2$$

Finally, the molecular density (i.e. the number of TMeOP units per unit cell) can be estimated as the ratio between N_{cells}, and Q_{mol} resulting in one molecule per few (3 ± 1) graphene unit cell for the case of the 5 mM solution.

This result is also consistent (within the approximation of the calculation) with the estimation obtained from the intensity ratio between the D and G bands in the Raman spectra[1]. In the high density (HD) condition[2], it is possible to estimate the inter defect distance L_D as:

$$L_D^2(HD) = 5.4x10^{-2}E_L^4(\frac{I_D}{I_G})$$

while in the low density (LD) condition[1]:

$$L_D^2(LD) = 4.3x10^3 E_L^{-4}(\frac{I_D}{I_G})^{-1}$$

where E_L is the laser photon energy (for He-Ne, E_L=1.96 eV) and I_D/ I_G the intensity ratio between the D and G Raman bands.

Considering that, as reported in the manuscript, after the electrochemical process the I_D/I_G becomes 0.92 for the treated sample and 1.4 for TMeOP-grafted one, we estimate L_D = (17.6 ± 2.3) nm for the treated in the low density regime and L_D = (1.07 ± 0.1) nm in the high density condition for the grafted sample.

The latter (L_D=1.07) yields a density of 1 molecule per 4.5 ± 0.5 graphene unit cell. This result is consistent with the molecular density estimated by the photoemission measurements. The assumption to use two different regimes for the treated and grafted sample was supported from the STM measurements carried out on the treated sample (Figure S4) where no defect seems to be present in the *11.5 x 11.5 nm²* STM image.

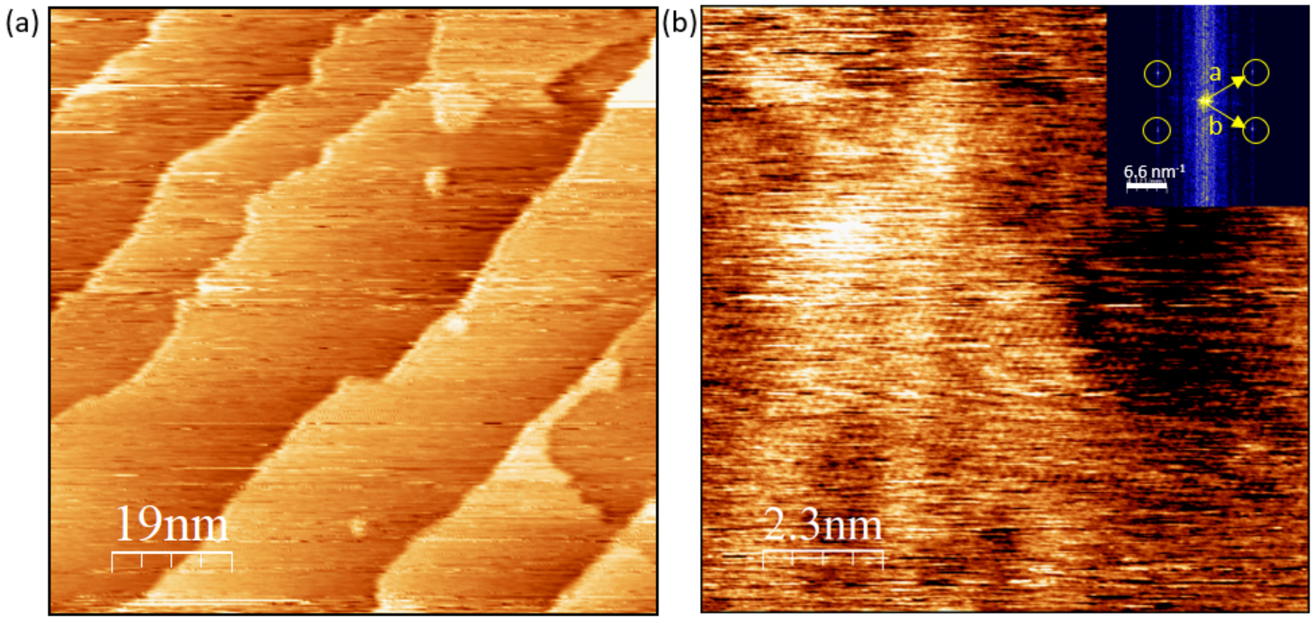

Figure S4: *STM images of treated sample at different image area: (a) 95 x 95 nm² (0.6 nA, - 1.7 V) and (b) 11.5 x 11.5 nm² (0.5 nA, - 0.035 V). The 2D-FFT reveals the symmetry and the dimension of the unit vectors of a = 0.56 nm⁻¹ and b = 0.55 nm⁻¹.*

As reported in the manuscript, the C 1s has been interpolated by considering the C sp[2], the O-C=O contamination, the π–π* shake up and for TMeOP -grafted graphene the contribution of the methoxy group (O-CH_3). To prove that the feature at 533.2 eV is due to the methoxy groups and not to oxygen contamination, in Figure S5 the C 1s and the O 1s XPS spectra for two different concentrations of the molecules (1 mM and 5 mM) in the electrochemical solution are shown. In both cases, the O-CH_3 contribution (purple peak in C 1s and orange peak in O 1s) increases with the molecule concentration, confirming that this feature could be taken as a fingerprint of molecule grafting.

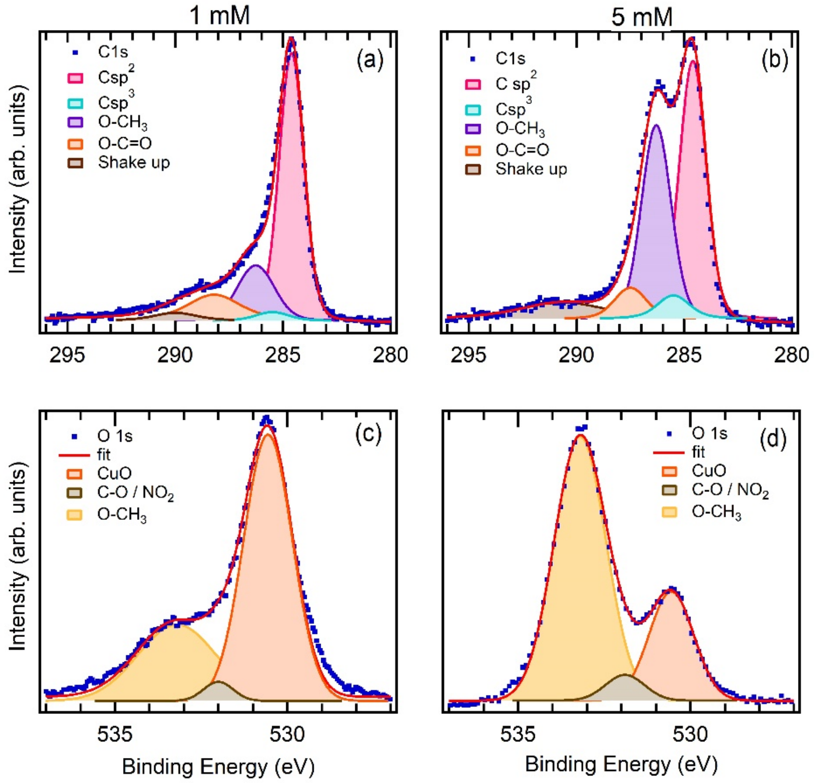

***Figure S5**: Core level spectra of TMeOP-grafted on Gr/Cu at different concentration. (a) C 1s and (c) O 1s spectra of 1 mM TMeOP-grafted on Gr/Cu(111). (b) C 1s and (d) O 1s spectra of 5 mM TMeOP-grafted on Gr/Cu foil.*

To understand the origin of the O 1s in the pristine graphene on copper, we consider the XPS spectra in the region of the O 1s XPS core level peak, at about 530 eV (Figure S6 a). The two structures observed in the spectrum are at 530.5 eV, ascribed to the CuO_x layer between graphene and the copper substrate, and at 532.5 and 533.9 eV, due to the air contamination (C-O) species and H_2O, respectively[3-6]. The origin of this peak has been proved by collecting the O 1s XPS spectrum also at 30° off-ormal emission, thereby increasing the surface sensitivity of the XPS probe. As expected, the ratio between the oxygen peaks at BE=532.5 eV and BE=533.9 eV (C-O and H_2O) and the component ascribed to CuO_x, duplicates with respect to the spectrum collected at normal emission (Figure S6 b).

Figure S7 shows the O 1s core levels collected on HOPG. All the samples on HOPG show a single structure. As expected, the O 1s intensity is negligible for all the samples except for TMeOP-grafted on HOPG where a clear O 1s core level is present at about 533 eV[7] due to the methoxy groups (or O-CH_3 groups). We note that the contribution at 530.5 eV, present on the spectra collected on Gr/Cu is

completely missing in HOPG samples, confirming that this oxygen component can be related to oxygen atoms placed between graphene and Cu. By comparing this value with the ones reported in literature[8] where the O 1s in CuO and Cu_2O is at 530.5 eV, in $Cu(OH)_2$+ OH^- at 532 eV and in H_2O at 533.0 eV, we can assume that the contribution at 530.5 eV comes from the Cu-O_x layer at the interface between graphene and copper[9].

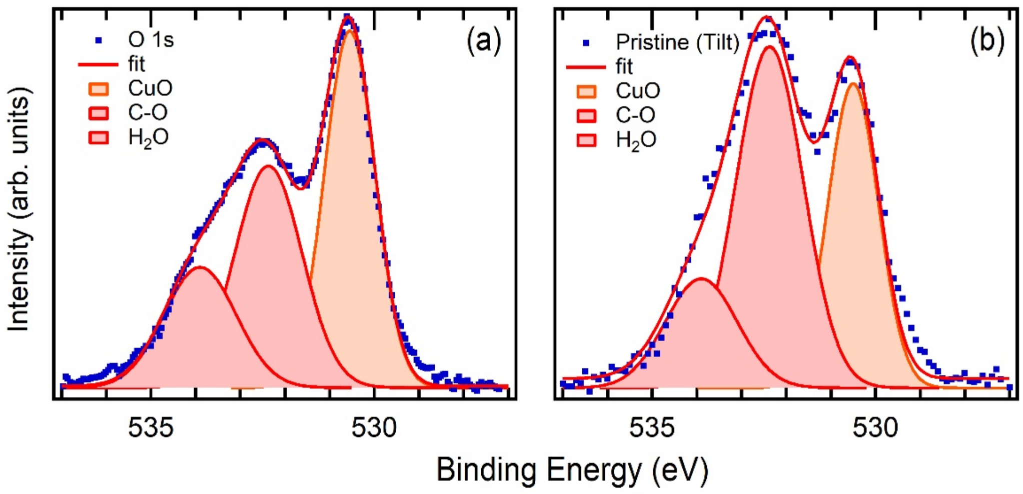

Figure S6: *O1s spectra on pristine Gr/Cu foil taken at (a) normal emission geometry and (b) with a tilt of 30° respect to the normal emission. The sample is pristine on Gr/Cu foil.*

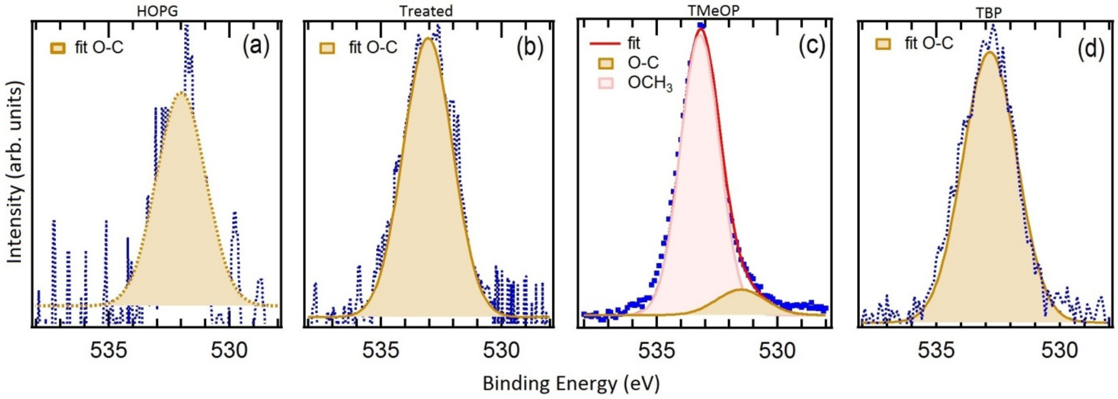

Figure S7: *XPS spectra of O1s core level on (a) HOPG, (b) treated HOPG, (c) TMeOP-grafted on HOPG, and (d) TBP-grafted on HOPG.*

Figure S8 shows the ARPES data acquired at the K point of the first Brillouin zone (BZ) of the pristine graphene on copper foil and of the sample grafted with 1 mM and 5 mM of TMeOP-grafted molecules on Gr/Cu(111) and Gr/Cu foil, respectively. In the pristine Gr/Cu foil, a Dirac cone appears downshifted with respect to the Fermi level, confirming that the CuO_x layer decouples the graphene from the substrate. Due to the high coverage, the ARPES image on 5mM of TMeOP-grafted Gr/Cu foil is completely dominated by the HOMO and HOMO-1 bands.

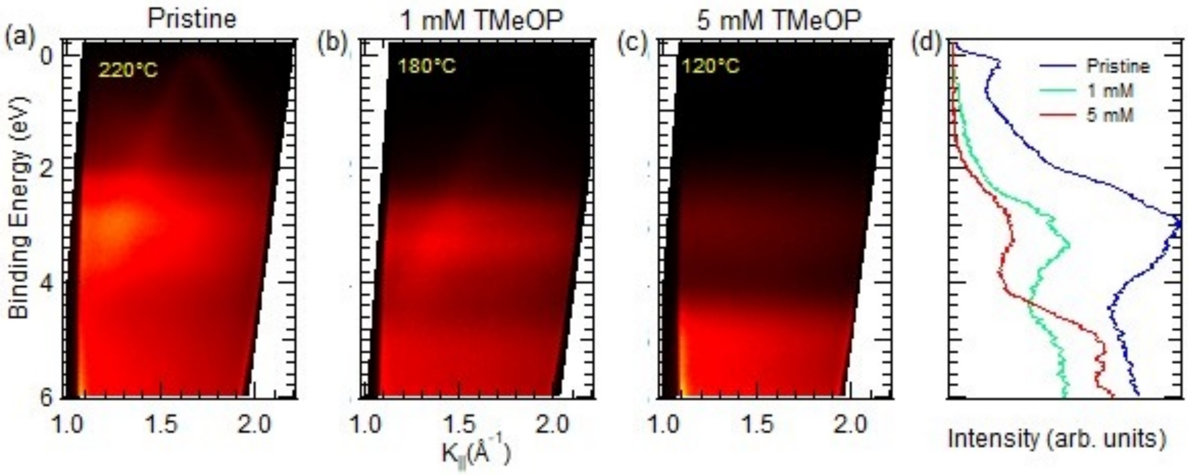

Figure S8: *ARPES measurements at the K-point of the first Brillouin zone on (a) pristine Gr/Cu foil, the image is obtained summing data in p and s polarizations, (b) on 1mM TMeOP-grafted on Gr/Cu(111), in p polarization, (c) on 5mM TMeOP-grafted on Gr/Cu foil in p polarization, and (d) corresponding energy distribution curves (EDCs) at* $k_{//}=1.7$ *Å*$^{-1}$*. The temperature of the annealing carried out before the ARPES acquisitions is also reported.*

We note that for this study, both Gr/Cu foil and Gr/Cu(111) have been used as a substrate for the grafting procedure even if, in principle, the two interfaces could be different. As previously discussed, the presence of Cu-O_x layer at the interface between graphene and copper, mainly due to the gentle annealing treatment, decouples the copper substrate from graphene, making the latter more free-standing and comparable the two interfaces. To further confirm this point, in Figure S9, the ARPES data collected at the Γ and the K-point of the first Brillouin zone are shown.

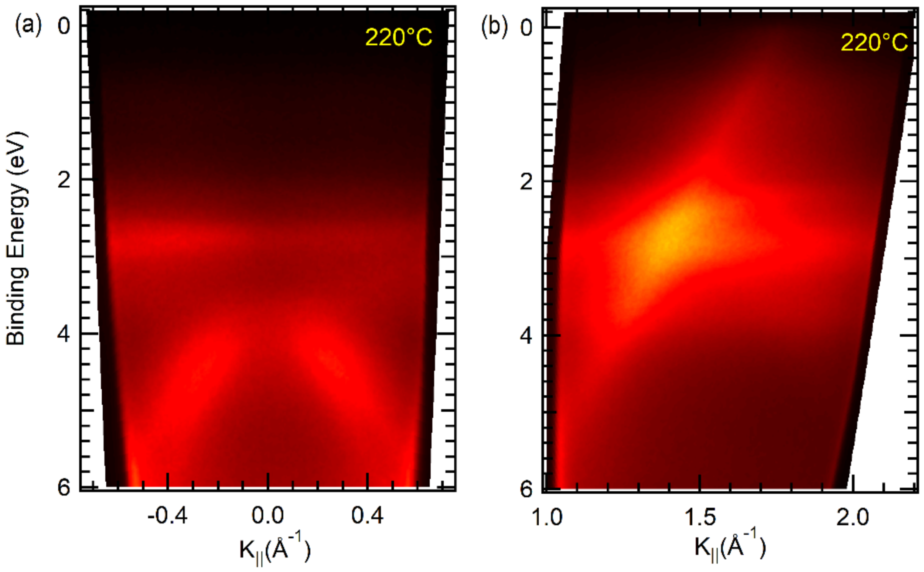

Figure S9: *ARPES measurements of Gr/Cu(111) at (a) Γ−point and (b) K-point of the first Brillouin zone in p polarization.*

At the Γ point, the Cu d band and the graphene σ band dispersion are at 2-4 eV and 4 eV, respectively, while the graphene Dirac cone appears at the K-point at the Fermi level as for a free-standing (undoped) graphene.

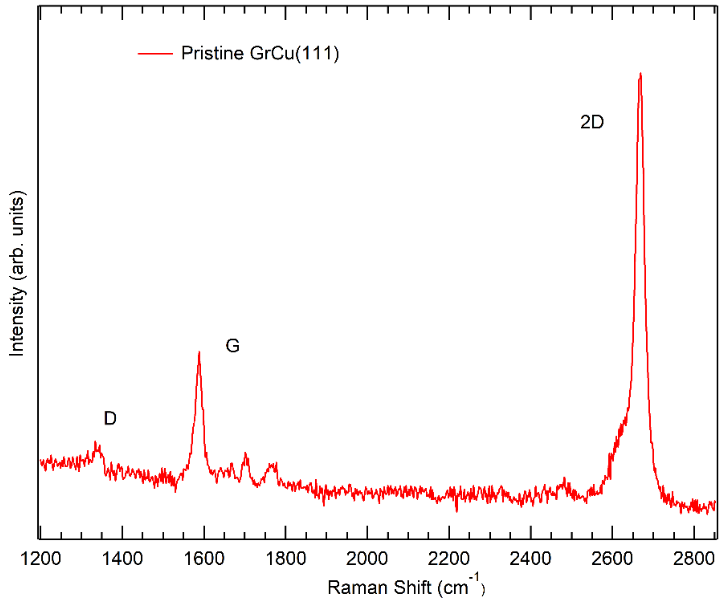

***Figure S10**: Raman spectrum of pristine Gr/Cu(111).*

Moreover, in Figure S10 the Raman spectrum collected on pristine Gr/Cu(111) is shown. The D/G band intensity ratio ($I_D/I_G = 0.07 \pm 0.04$) is similar to that of Gr/Cu foil ($I_D/I_G = 0.105 \pm 0.06$ as shown in Figure 2 of the manuscript) confirming that the quantity of defects and the degree of disorder of the graphene layers grown on both interfaces are comparable.

REFERENCES

1. Cançado, L.G. et al. Quantifying defects in graphene via Raman spectroscopy at different excitation energies. *Nano Lett.* **11**, 3190-6 (2011).
2. Ghosh, S. et al. Evolution and defect analysis of vertical graphene nanosheets. *J. Raman Spectrosc.* **45**, 642-649 (2014).
3. Hurley, B.L. & McCreery, R.L. Covalent Bonding of Organic Molecules to Cu and Al Alloy 2024 T3 Surfaces via Diazonium Ion Reduction. *Journal of The Electrochemical Society* **151**, B252 (2004).
4. Akhavan, O., Azimirad, R., Safad, S. & Hasanie, E. $CuO/Cu(OH)_2$ hierarchical nanostructures as bactericidal photocatalysts. *J. Mater. Chem.* **21**, 9634-9640 (2011).
5. Pawar, S.M. et al. Multi-functional reactively-sputtered copper oxide electrodes for supercapacitor and electro-catalyst in direct methanol fuel cell applications. *Sci. Rep.* **6**, 21310 (2016).
6. Kidambi, P.R. et al. Observing graphene grow: catalyst-graphene interactions during scalable graphene growth on polycrystalline copper. *Nano Lett.* **13**, 4769-4778 (2013).
7. Zhao, C.X., Zhang, Y., Deng, S.Z., Xu, N.S. & Chen, J. Surface nitrogen functionality for the enhanced field emission of free-standing few-layer graphene nanowalls. *Journal of Alloys and Compounds* **672**, 433-439 (2016).
8. Akhavan, O. Chemical durability of metallic copper nanoparticles in silica thin films synthesized by sol-gel. *J. Phys. D: Appl. Phys.* **41**, 235407 (2008).
9. Gottardi, S. et al. Comparing graphene growth on Cu(111) versus oxidized Cu(111). *Nano Lett.* **15**, 917-922 (2015).
